# Prediction of Microscopic Metastases in Patients with Metachronous Oligo-Metastases after Curative Treatment of Non-Small Cell Lung Cancer: A Microsimulation Study

**DOI:** 10.3390/cancers13081884

**Published:** 2021-04-14

**Authors:** Henri B. Wolff, Leonie Alberts, Elisabeth A. Kastelijn, Naomi E. Verstegen, Sherif Y. El Sharouni, Franz M. N. H. Schramel, Rein Vos, Veerle M. H. Coupé

**Affiliations:** 1 Department of Epidemiology and Data Science, Amsterdam Public Health Research Institute, Amsterdam UMC, Location VUmc, 1081 HV Amsterdam, The Netherlands; v.coupe@amsterdamumc.nl; 2 Department of Pulmonology, St. Antonius Hospital, 3435 CM Nieuwegein, The Netherlands; l.alberts@antoniusziekenhuis.nl (L.A.); l.kastelijn1@antoniusziekenhuis.nl (E.A.K.); f.schramel@antoniusziekenhuis.nl (F.M.N.H.S.); 3 Department of Radiation Oncology, Amsterdam UMC, Vrije Universiteit Amsterdam, 1081 HV Amsterdam, The Netherlands; nverstegen@spaarnegasthuis.nl; 4 Department of Radiation Oncology, University Medical Centre Utrecht, 3584 CX Utrecht, The Netherlands; S.Y.ElSharouni@umcutrecht.nl; 5 Department of Methodology & Statistics, Maastricht University Medical Centre, 6200 MD Maastricht, The Netherlands; rein.vos@maastrichtuniversity.nl

**Keywords:** oligo-recurrence, non-small cell lung cancer, prediction model

## Abstract

**Simple Summary:**

Many patients with metachronous oligo-metastases in non-small cell lung cancer have their recurrences surgically removed, although the 5-year recurrence-free survival of this group is 16%. This does not provide any benefit for patients with additional undetected metastases. Therefore, we aim to find patient characteristics that are predictive for having additional undetected microscopic metastases. Based on a theoretical approach, we identified the size and number of detected oligo-metastases, as well as the presence of symptoms that are the most important risk predictors.

**Abstract:**

Metachronous oligo-metastatic disease is variably defined as one to five metastases detected after a disease-free interval and treatment of the primary tumour with curative intent. Oligo-metastases in non-small cell lung cancer (NSCLC) are often treated with curative intent. However additional metastases are often detected later in time, and the 5-year survival is low. Burdensome surgical treatment in patients with undetected metastases may be avoided if patients with a high versus low risk of undetected metastases can be separated. Because there is no clinical data on undetected metastases available, a microsimulation model of the development and detection of metastases in 100,000 hypothetical stage I NSCLC patients with a controlled primary tumour was constructed. The model uses data from the literature as well as patient-level data. Calibration was used for the unobservable model parameters. Metastases can be detected by a scheduled scan, or an unplanned scan when the patient develops symptoms. The observable information at time of detection is used to identify subgroups of patients with a different risk of undetectable metastases. We identified the size and number of detected oligo-metastases, as well as the presence of symptoms that are the most important risk predictors. Based on these predictors, patients could be divided into a low-risk and a high-risk group, having a model-based predicted probability of 8.1% and 89.3% to have undetected metastases, respectively. Currently, the model is based on a synthesis of the literature data and individual patient-level data that were not collected for the purpose of this study. Optimization and validation of the model is necessary to allow clinical usability. We describe the type of data that needs to be collected to update our model, as well as the design of such a validation study.

## 1. Introduction

Metachronous oligo-metastatic disease is variably defined as one to three or one to five metastases detected after a progression-free interval and a controlled primary tumour [1,2,3,4]. Oligo-metastases in non-small cell lung cancer (NSCLC) are often treated with curative intent, but additional metastases are commonly detected later in time, and the 5-year survival is low in this group of patients [5]. These patients are assumed to have had poly-metastatic disease at time of the oligo-recurrence, with most metastases below the detection threshold of the scan. In this paper, we will refer to oligo-metastatic disease with additional undetected metastases as “oligo+”, and without undetected metastases as “oligo−”. Currently, clinicians have no tools available that can distinguish “oligo+” from “oligo−” patients [6,7,8].

Curative therapy is likely to provide limited benefit in oligo+ patients [9]. Furthermore, such therapies can result in adverse events or even treatment-related mortality [10,11]. Therefore, it is vital to develop tools to distinguish between oligo+ and oligo− patients; that is, identify the predictors that would allow for such a distinction.

There are two important obstacles for the development of such a tool. Firstly, the true outcome of interest is the presence of unobserved metastases, an outcome that is by definition unknown (Box 1). Therefore, most studies use surrogate endpoints, such as prognostic factors, for survival [12,13,14]. These studies include factors such as the patients’ age and performance score into their models, which may select a subgroup of both curable and incurable patients that live relatively longer, or administration of chemotherapy. Those factors are good predictors when the focus is on prognosis in a mixed group of patients with oligo+ and oligo−.

A second obstacle is that there are difficulties with patient accrual in randomized controlled trials [15]. Currently, most studies on oligo-metastases are retrospective studies, with small groups of patients. As a result, these studies are susceptible to selection bias, and the levels of evidence are often weak [16,17,18,19].

To tackle both obstacles, we have chosen to construct a microsimulation model of the development and detection of metastases in stage I NSCLC patients. Microsimulation models allow simulation of detailed disease trajectories of a large number of individual patients based on a mathematical model describing how patients transition between health states. Microsimulation models have the advantage that all available evidence can be synthesized to calibrate unobservable parameters, such as the presence of undetectable metastases. In our model, curatively treated patients may develop a number of metastatic lesions that grow exponentially over time. The microsimulation model keeps track of the growth all metastases within one patient, the number of metastases detected either by surveillance or by symptoms, and the recurrence-free survival.

We used a microsimulation model to construct a simulated patient-level dataset, in which individuals were classified as having detected oligo-metastases or poly-metastases, with the former being subdivided into an oligo− and oligo+ group. Subsequently, we used the simulated dataset to identify the clinically observable patient characteristics that can predict the presence of undetected metastases.

We describe the development of the microsimulation model and the underlying evidence. In addition, we present the simulated dataset and the identification of the observable patient characteristics that are related to the risk of having undetected recurrences. Finally, we discuss the requirements for future validation of our findings and identification of additional predictors, such that the identification of patients that benefit from curative treatment of oligo-metastases may be improved.

Box 1 **Definitions used.**

**Curative treatment:** Treatment focused on removing or destroying tumours locally, with the aim to make the patient disease free.

**Metastases:** All tumours originating from the primary tumour or another metastasis.

**Recurrences:** All metastases detected on a scan.

**Oligo-metastasis:** Several definitions have been used in the literature. In this paper, oligo-metastasis is defined as three or less detected recurrences within one patient, with a controlled primary tumour.

**Poly-metastasis:** Several definitions have been used in the literature. In this paper, poly-metastasis is defined as four or more metastases detected within one patient.

**Metachronous oligo-metastasis:** The oligo-metastasis is detected after curative treatment of the primary tumour, contrary to synchronous oligo-metastasis.

**Undetectable metastases:** In this paper, all (microscopic) metastases of a size below the detection threshold.

**RFS:** Recurrence-free survival—the proportion of patients that remain recurrence-free after curative treatment of the primary tumour.

**PFS:** Progression-free survival—the proportion of patients that have no additional recurrences detected after curative treatment of oligo-metastases.

**oligo+:** A patient with oligo-metastasis(es) with additional undetectable metastases, who would be classified as poly-metastatic if the true number of metastases would be known.

**oligo−:** A patient with oligo-metastasis(es) without additional undetectable metastases.

**VDT:** Volume doubling time—the time required for the tumour to double in volume.

## 2. Materials and Methods

### 2.1. Concept of the Microsimulation Model

**Figure 1:**
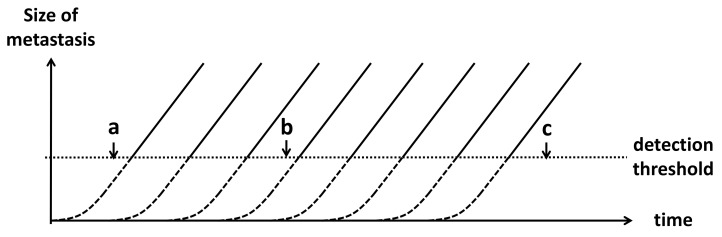
Growth of metastases in time. Metastases are considered undetectable when their size is below the detection threshold (dashed lines), and thus become detectable above the detection threshold (solid lines). If a patient is scanned at time “a”, all metastases are invisible, and this patient would be considered to be “recurrence-free”. At time “b”, three recurrences would be visible on the scan. This is defined as an oligo-metastasis, even though there are several metastases under the detection threshold (oligo+). At time “c”, 8 recurrences are visible on the scan, which is defined as poly-metastatic disease.

Curative treatment of oligo-metastases has a pooled 16% 5-year progression-free survival (PFS) [6,7,8,20,21]. This low PFS is assumed to be caused by undetected metastases that already existed at time of treatment of the primary tumour (Figure 1). The purpose of the microsimulation model is to test which observable patient characteristics during detection of oligo-metastases may be predictive for the presence of undetected metastases, by synthesizing the available evidence on the growth of tumours, recurrence patterns, and methods of detection using the current theory and available data.

The microsimulation model was developed in C++ and describes the growth and detection of metastases in individual patients. The model stores all patient-specific features and outcomes in comma-separated values files for further analysis. Microsoft Excel Professional plus 2016 was used to create the figures.

Certain assumptions and simplifications had to be made, to allow modelling of cancer progression and metastases, without making the model overly complex for the research question that we aim to address (Box 2). These model assumptions have been tested in the sensitivity analyses. The model contains both directly observable parameters as well as unobservable parameters. The observable parameters were directly estimated based on patient data and the literature (Table 1). Calibration against the observable output variables was used to estimate the unobservable (hidden) parameters, such as the number of undetectable metastases (Table 1).

Box 2 **Model assumptions.**

The most important assumptions of the microsimulation model are listed below, as well as the reasoning behind these assumptions.

1. An exponential tumour growth model using volume doubling time was chosen, because volume doubling time is the most commonly used statistic in the literature. All metastases within one patient are assumed to have the same growth rate, and are formed consecutively with fixed time intervals. This assumption has been tested in Scenario 2 of the sensitivity analyses.
2. Each patient is assumed to have a fixed number of metastases after their primary tumour had been curatively treated. The proportion of patients that have zero metastases after curative treatment equals the proportion of patients that are recurrence-free after 5 years. In all other patients, the number of metastases is randomly drawn from a rounded truncated normal distribution.
3. We assume that metastases below the minimum detectable size for the Computed Tomography scan will always be missed.
4. Metastases of detectable size are either found during surveillance or on an unscheduled scan because of symptoms, whichever happens first. Other scenarios are not considered. Surveillance CT scans are able to detect recurrences to the lung, liver, and adrenal glands. Bone and brain metastases are highly symptomatic. Less than 3% of NSCLC metastases are found in other organs, and these metastases are often also symptomatic [22,23]. Therefore, we assume that the com-bination of symptomatic and surveillance detection sufficiently describes the detection patterns.
5. Once one recurrence is detected, a more rigorous examination, that is, Positron Emission Tomography–Computed Tomography, follows, resulting in detection of all metastases above the minimum detectable size.
6. The proportion of patients with microscopic metastases within those with detected oligo-recurrent disease is assumed to be equal to the proportion of patients with a 5-year PFS after treatment of oligo-recurrent disease. This may lead to an underestimation of the proportion of oligo-metastases. Therefore, this assumption was further investigated in Scenario 1 of the sensitivity analyses.

### 2.2. Model Functions 

Each simulated patient starts with a number of metastases (*M_total_*) drawn from a truncated normal distribution, *N*(*σ,μ*) ≥ 1, rounded up to integer numbers. All metastases in the model grow with a patient-specific volume doubling time (*VDT*) in days^−1^ [35].

(1) $$V_{m}\left(t\right)=V_{m}\left(0\right)\;\times \;2^{t/VDT}$$

Here, *V_m_*(*t*) is the volume (in cm^3^) of metastasis *m*, *m* = 1… *M_total_*, at time *t* (in days). The size of the largest metastasis is used to calculate the sizes of the other metastases. The relative sizes of the metastases are defined by the size ratio R [36]:

(2) $$V_{m}\left(t\right)=V_{0}\left(t\right)\;\times \;R^{m}$$

In the model, metastases can either be detected during a routine surveillance scan, or when the metastases become symptomatic. Metastases become detectable on a CT scan once their size is equal or larger than the minimum detectable volume, *V_det_*. An exponential hazard function for becoming symptomatic is assumed:

(3) $$P_{m}\left(symptom\;|\;t\right)=\left\{\begin{array}{c} 0\\ \lambda_{symptom}e^{-\lambda_{symptom}\;\left(t-t_{\det m}\right)} \end{array}\right.\begin{array}{c} \;t<t_{\det m}\\ \;t\geq t_{\det m} \end{array}$$

with *λ_symptom_* the hazard to develop symptoms. Time *t*_det m_ is defined such that $V_{m}\left(t_{\det m}\right)=V_{det}$. Metastases smaller than *V_det_* are assumed to be too small to cause symptoms.

### 2.3. Parameter Estimation

The model parameters have been directly estimated from patient data and the literature when possible. All the unobservable parameters were estimated using calibration in combination with other observable target parameters. 

Data of stage I NSCLC patients curatively treated with video-assisted thoracoscopic surgery and stereotactic body radiation therapy were obtained from two studies performed in the Netherlands between 2003 and 2013 [24,25]. Patients were excluded if they had ≥stage II disease, an ECOG performance score ≥2, a second primary tumour, or history with previous cancer. Patients of both studies were pooled and their 1:1 propensity score matched on treatment using the Matching R package, version 4.9-2 [37]. Recurrence-free survival (RFS) was analysed with a Kaplan–Meier survival curve using Statistical Package for Social Sciences version 22.0 [38]. 

**Figure 2:**
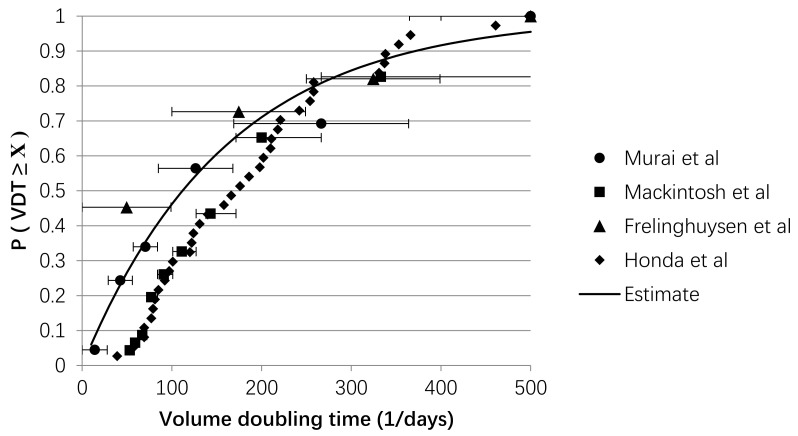
Estimation of the cumulative distribution function of the volume doubling time (*VDT*). *VDT* values found in literature were used to create the scatterplot [26,27,28,29]. A cumulative distribution function is fitted using a least square function to the data shown in this scatterplot to obtain a quantile function, which was subsequently used to determine the random tumour growth rates per patient.

The VDTs were obtained from papers that reported the distribution of the growth rates measured with CT equipment, in NSCLC with mixed histologies and a non-specific tumour morphology [26,27,28,29]. Some papers classified growth rates into groups (fast, moderate, slow, and no growth). For these groups, the average of the minimum and maximum growth rates was assumed to be the average VDT of the reported group. A cumulative exponential distribution was fitted to the reported data using a least squared estimate function (Figure 2). This distribution was used to sample the VDTs for patients in the microsimulation model.

Smaller metastases are more likely to be missed on a scan [39,40]. As such, the reported scan sensitivities and specificities for tumours of any size as found in the literature are not suitable for our continuous tumour growth microsimulation model [41]. Instead, we have therefore chosen to use a detection threshold. We assumed a detection threshold of 5 mm diameter on the CT scans [31,32], corresponding to a spherical volume of 0.07 cm^3^.

### 2.4. Model Calibration

Four (sets of) parameters needed to be calibrated: (1) the detection rate parameter *λ_det_*, which is used to randomly draw the time when the largest metastasis passes the detection threshold; (2) the parameters used to determine the total number of metastases per patient *M_total_*(*µ*,*σ*); (3) the parameters used to determine the size ratio of the metastases *R*(*α,β*); and (4) the hazard of a metastasis becoming symptomatic (*λ_symptom_*). Each of these model parameters is strongly associated with a specific model output parameter that is also available from the literature or patient-level data (see below). The mean squared error of 1000 simulations was used in combination with a univariate or grid search algorithm (for *M_total_*(*µ*,*σ*), and *R*(*α*,*β*)) and repeated until the calibration target was matched up to 3 significant figure places [42,43].

Calibrated parameters only interact in one direction: the number of metastases influences the chance that a patient becomes symptomatic, but the hazard of a single metastasis becoming symptomatic does not influence the number of metastases per patient. Therefore, parameters could be calibrated consecutively if done in the correct order—the most dominant parameter first.

Firstly, the distribution of the time to detectability (that is, reaching a 5 mm diameter) of the largest metastasis was assumed to be a negative exponential distribution $\left(t_{det}=\frac{ln\left(rand\right)}{\lambda_{det}}\right)$. *λ_det_* was calibrated against the ½ yearly RFS, weighted for the number of patients at risk. Patient-level data of the two retrospective Dutch cohort studies described above were pooled to obtain the RFS statistics [24,25]. Both the simulations and the patient-level data used the same surveillance schedule of the Dutch guidelines [44].

Secondly, *M_total_* represents the number of both detected and undetectable metastases per patient. There are no data available on this number, nor on the distribution of the detected metastases. Therefore, *M_total_* is assumed to be normally distributed, but is truncated such that *M_total_* ≥ 1. *M_total_*(*µ*,*σ*) was calibrated against the proportion of oligo-metastases without undetected metastases. To determine this proportion, it was assumed that patients who are curatively treated for oligo-metastases and are progression-free at 5 years do not have undetected metastases. A weighted average of the estimates reported in the medical scientific literature was calculated, resulting in an estimated 16% of oligo-metastases that have no underlying undetected metastases [6,7,8,20,21]. To allow a unique solution for *µ* and *σ*, we added to following constraint: the solution for µ and σ that minimizes the maximum number of detected metastases under the current surveillance schedule.

Thirdly, the size ratio of the metastases, R, is calculated as the volume of the second largest metastasis divided by the volume of the largest metastasis. Therefore, R has a value between 0 and 1 by definition. We used a Beta distribution to draw the *R* for each patient. The shape parameters *α*,*β* were defined as integer values. *R*(*α*,*β*) was calibrated against the pooled average proportion of oligo− [7,20,21]. As a constraint, the solution with the smallest lower tail was selected.

The last calibrated variable was *λ_symptom_*, the hazard of a single metastasis becoming symptomatic. *λ_symptom_* was calibrated against the ratio of the symptomatically (or unscheduled) detected metastases to metastases detected by a scheduled scan [33,34].

### 2.5. Model Simulations

The life histories of 100,000 patients with stage I NSCLC and one or more undetected metastases were simulated from treatment of the primary tumour until the detection of a metastasis (RFS). Patients without micro-metastatic disease after curative treatment of the primary tumour were not simulated but were added to the cohort post-simulation in accordance with the proportion of patients that were recurrence-free after 5 years.

For the simulation, each individual was randomly generated from the fitted distributions: the total number of metastases *M_total_*, the size ratio R of the metastases, the volume doubling time, the time that the metastases would grow past the detection threshold, *t*_det_, and the time that the patient would develop symptoms, *t_symp_*. Subsequently, the model determines if symptomatic detection occurs before or after the time that the tumour would be detected on a surveillance scan. Once the time of detection is determined, the number of metastases visible on a scan *m_det_* can be calculated with Function (4). Function (4) can be derived from Functions (1) and (2) (Appendix A.1). The point in time that the largest metastasis becomes detectable on a scan is denoted by *t*_det_ = 0.

(4) $$m_{det}=\lceil \frac{\left(t_{\det0}-t_{scan}\right)}{VDT\times log_{2}\left(R\right)}\rceil .$$

### 2.6. Prognostic Groups

A binary logistic regression model was used for the purpose of identifying the clinically observable predictors that can distinguish oligo− from oligo+. The parameters that were considered clinically observable were the number of recurrences detected; the size category of the largest detected recurrence: small (<6 mm), medium (6–8 mm), or large (>8 mm) [32], symptomatic or surveillance detection; and the RFI (years) [12]. All predictive covariates were tested for multi-collinearity by calculating the variance inflation factor (VIF) for each variable. A residual and a binned residual plot for the logistic regression analysis was made to test if the assumptions of the logistic regression model were met.

Predictors with a Wald statistic *p* < 0.05 were used to divide the simulated patients into prognostic sub-groups and determine the proportion of oligo+ for each of these groups. These sub-groups were pooled into a low-risk group with less than 30% oligo+, and a high-risk group with more than 30% oligo+. The 30% risk threshold was considered an acceptable level to consider curative therapy, based on a discussion with our clinical experts (LA, EAK, SYS, and FMNHS). Still, this 30% threshold is suggested tentatively, as it is not obtained from any formal consensus procedure. However, the threshold allows for accessible presentation of the performance and potential use of our modelling approach, and can be seen as a first step towards decision support in this area.

### 2.7. Sensitivity Analyses

To test the robustness of our results, we have designed a series of scenarios and tested how much the predictors used to determine the risk groups, proportion of oligo+, and size of the risk groups are affected by these scenarios.

1. Recalibration of model parameters to the upper and lower confidence interval of their targets.
2. Random variation in VDT of metastases within one patient and in the detection threshold.
3. Correlation between the volume doubling time and the total number of metastases per patient.
4. Redefinition of the oligo-metastases threshold to 1 or to 5 metastases.
5. The ability of the metastases to produce new metastases.

All sensitivity analysis scenarios are described in detail in Appendix A.

## 3. Results

### 3.1. Calibration

An overview of all the calibrated parameters can be found in Table 1.

**Figure 3:**
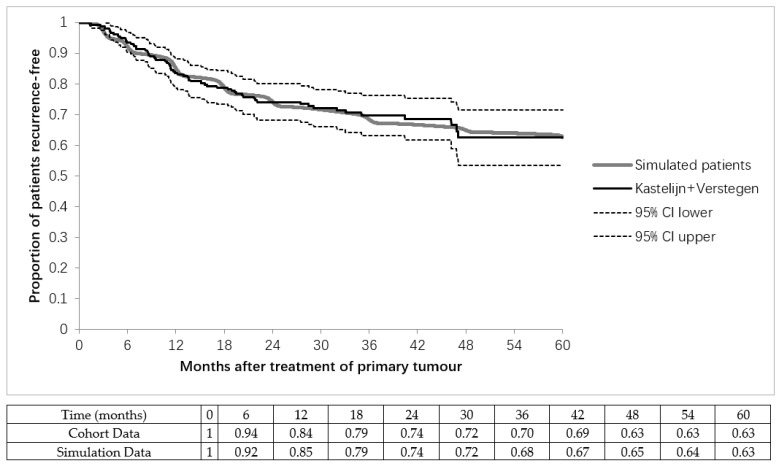
Detection of recurrences. Recurrence-free survival after curative treatment of the primary tumour of the simulated patients is calibrated to match two Dutch cohorts [24,25]. Recurrences are detected with a surveillance scan schedule and unplanned additional scans using a symptom hazard model. A total of 62.5% of the Dutch cohorts remained recurrence-free. Patients without recurrences were not simulated but were added after the simulation for the purpose of showing the differences between the simulated patients and the Dutch cohorts in this figure. Number of detected recurrences per month. Asymptomatic recurrences were only detected during surveillance scans. These scans were planned according to a fixed schedule, with a small variation in scan date N (*μ* = FU schedule, *σ* = 15.5). Symptomatic recurrences were detected throughout the year, and detection times were determined with the hazard model (Function (3)).

Figure 3 shows the resulting model-based progression-free survival curve compared to the two Dutch cohorts [24,25]. The simulated progression-free survival is within the 95% CI range of the cohort data with the exception of the first and third month, but the 95% CI was never exceeded by more than 1.1% after calibration of *λ*_detectable_. 

Calibration of *M_total_* resulted in a maximum of 70, an average of 9, and a median of 6 metastases detected. This solution was considered realistic, based on discussion with our clinical experts LA, EAK, SYS, and FMNHS.

Calibration of the size ratio *R* resulted in *α* = 9 and *β* = 1, giving an average *R* of 0.93. 

The proportion of symptomatically detected metastases was 33.5% after calibration of the parameter *λ_symptom_*, similar to the proportion observed in clinical practice [33,34].

### 3.2. Simulation Results

The microsimulation model was used to produce a patient-level database of 100,000 patients. The first row of Table 2 shows the characteristics for all the simulated patients. These patients are subdivided into three subgroups in row two to four: poly-metastases (patients with 4 or more detected metastases), oligo+ (patients with 3 or less detected metastases and additional undetectable metastases), and oligo− (patients with 3 or less detected metastases and no additional undetectable metastases). Between these three subgroups, the ranges for all the reported characteristics are largely overlapping, although the skewness of the distributions is different. The median oligo+ patient has a large size ratio between the metastases, slow-growing metastases, small diameters at the time of detection, and a low RFI.

### 3.3. Prognostic Groups

**Figure A1:**
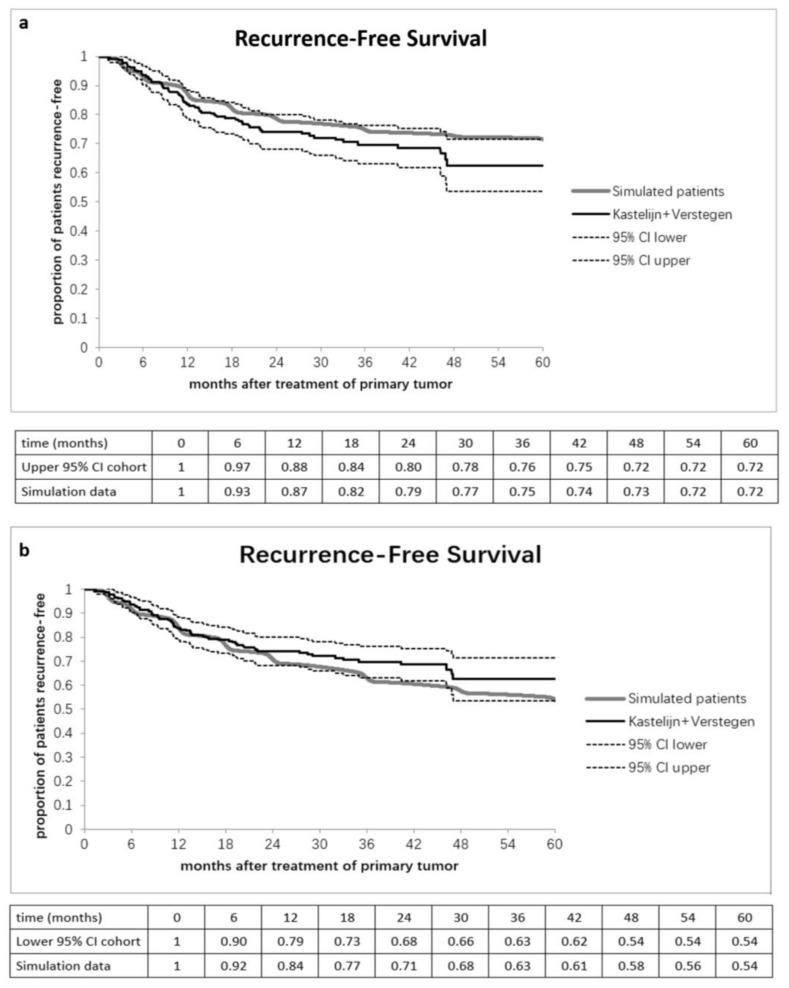
Detection of recurrences in sensitivity analyses. Sensitivity analysis with t detectable calibrated to upper 95% CI of the progression-free survival (**a**) and the lower 95% CI of the progression-free survival (**b**).

The VIF for the predictors in the logistic regression model ranged from 1.016 to 1.097, and no transformation of variables were assumed to be needed based on the residual and binned residual plots (Figure A1). Although there is a large difference in the median RFI of the oligo+ and oligo− groups, as shown in Table 2, RFI is not a significant predictor in the logistic regression model (Table 3), when the size of the largest metastasis is included in the model. All the other predictors (size, number of detected metastases, and symptomatic detection) are significant, and are therefore used to construct Table 4.

A logistic regression model was used as an explorative search for the parameters suitable to determine the risk groups. Odds Ratios are calculated as exp(*β*). Year of detection was removed from the regression model because it was not a significant predictor (Wald test *p* > 0.05). All other covariates were significant (Wald test *p* < 0.0001). Note that the significance is influenced by the large number of simulated patients.

Table 4 shows the proportion of oligo+ at time of detection of recurrence within each of the subgroups defined by the three identified predictors. The 30% oligo+ threshold resulted in two distinct groups of patients. Patients with a large-sized metastasis or a single medium-sized metastasis fall in the low-risk group, and patients with small or 2 or 3 medium-sized metastases fall in the high-risk group. Although the symptoms do have an effect on the proportion of oligo+, this effect is not large enough to differentiate between low and high risk.

The pooled low-risk group has a proportion of 8.1% oligo+, and the high-risk group has a proportion of 89.3% oligo+. As shown in Table 2, the differences between the high- and the low-risk groups are larger than the differences between oligo+ and oligo−. This is a result of the selection procedure for the risk groups. The high- and low-risk groups were compared to the “treat-all” and “treat-none” strategies on their strategy performance (Table 5). In total, 86% of the patients with an oligo-recurrence are in the oligo+ group, which is the cause of the difference in accuracy between “treat-all” and “treat-none”.

**Figure A2:**
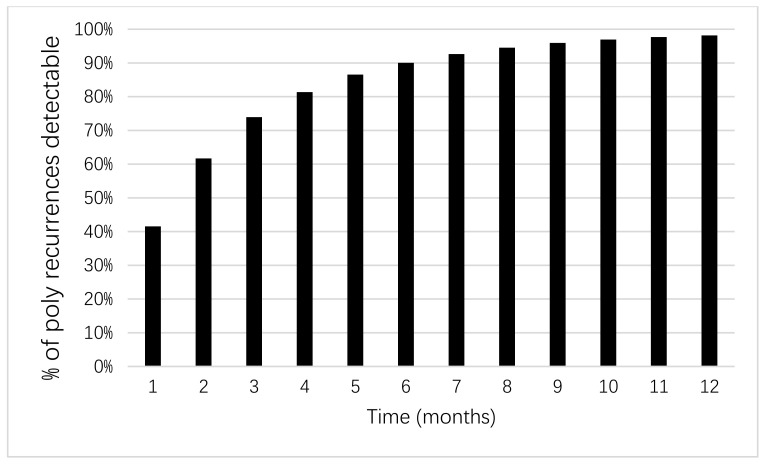
Time required for oligo+ to become distinguishable as poly-recurrent disease. This is estimated as the time that the 4th metastasis passes the detection threshold. 73.8% of oligo+ metastases become detectable after 3 months, and 98.2% of the oligo+ becomes detectable after one year. Data is based on the extrapolation of the microsimulation-model.

Additional analysis of the simulation output by extrapolation of the growth model of metastases showed that 73.8% of the oligo+ patients would switch to detectable poly-recurrent disease within 3 months, and 98.2% would become detectable within one year (Figure A2).

### 3.4. Sensitivity Analyses

A summary of all the sensitivity analyses is given in Table 6. Details of the proportion of oligo+ per subgroup are shown in Table A1, Table A2, Table A3, Table A4, Table A5, Table A6, Table A7, Table A8, Table A9, Table A10, Table A11, Table A12, Table A13, Table A14, Table A15, Table A16 and Table A17. The model output, such as the diameters of the metastases, or RFI, remained within realistic ranges under all scenarios.

The classification into two risk groups and the proportion of oligo+ in the low- versus high-risk group were similar to our initial analysis in those scenarios in which the model parameters were recalibrated (Scenarios 1–8). A change in the proportion of oligo+ in all simulated patients was associated with a change in the proportion of oligo+ in the risk groups. This was the most extreme for scenarios in which the *M_total_* and *R* varied (Figure A1).

Random variation in the detection size and growth rates of the metastases within one patient only caused a small increase in oligo+ patients in the low-risk group (Scenarios 9 and 10).

In Scenarios 11 and 12, in which the model parameters were correlated, the proportion of oligo+ in the low-risk group decreased even though the size of the low-risk group increased. Simultaneously, the proportion of oligo+ in the high-risk group increased. Thus, the ability to separate low- from high-risk patients increased with the correlated model parameters.

When oligo metastases are defined as 1 metastasis or as 1 to 5 metastases, this change in definition directly affects the total number of patients considered for curative treatment of their oligo-metastases. Furthermore, the number of detected metastases is a predictor that determines if a patient is high risk or not (Table A13 and Table A14). Using the definitions of oligo-metastasis as a single detected metastasis results in a risk model with overall better accuracy, but also a smaller number of patients that is considered to have an oligo-recurrence.

Metastatic metastases had a large direct effect on the proportion of oligo+, resulting in a significant increase in the proportion of oligo+ in all subgroups. As such, more subgroups passed the 30% oligo+ threshold, resulting in fewer subgroups to be pooled into the low-risk group. There are no patients with an acceptable risk to undergo curative therapy left, if 80% of all undetectable metastases are metastasizing at the time of curative treatment. Because a significant amount of patients do survive 5 years without additional recurrences after curative treatment of oligo-metastases in clinical practice, it is likely that the number of metastasizing metastases is low [6,7,8,20,21].

Over the different sensitivity analyses, the proportion of oligo+ at the time of detection of an oligo-metastasis remained relatively stable and resulted in acceptable average risks for curative therapy in the low-risk group. Furthermore, the combinations of predictors that were pooled into the low-risk group remained stable with the exception of the metastatic metastases scenarios.

## 4. Discussion

### 4.1. Key Findings

A microsimulation model of the growth and detection of metastases in individual patients was built based on what we considered to be realistic assumptions and an appropriate level of complexity. The model is able to reproduce detection patterns of metastases as seen in the clinical setting, based on the growth rates and other model parameters obtained from the literature. The model output was used to identify the characteristics that allow prediction of the presence of microscopic disease; that is, the presence of additional undetectable metastases in patients with oligo-metastases. Based on three selected risk predictors, the population of patients with an oligo-recurrence could be grouped into a small low-risk group with an 8.1% risk and a high-risk group with an 89.3% risk of microscopic disease. Sensitivity analyses showed that the model is robust to changes in parameters.

### 4.2. Clinical Implications

Currently, many stage I NSCLC patients in whom oligo-metastatic disease is detected are curatively treated for these oligo-metastases, although only 16% of those patients remain progression-free for 5 years [6,7,8,20,21]. For these patients, curative treatment provides no benefit but does provide additional risks and side-effects. Multiple prognostic factors for patients with oligo-metastases have been identified, but most of these predictors, such as usage of adjuvant chemotherapy, are not suitable to predict the presence of undetected metastases and do not allow for selecting those patients that potentially benefit from curative treatment [12]. The key novelty in this paper is that we study the underlying tumour growth to predict which patients are most likely to have additional undetected metastases, rather than identifying the prognostic factors for survival.

Current guidelines suggest that curative treatment for oligo-metastases can be considered [44]. As a result, there is considerable variation in treatment of oligo-metastases in clinical practice. As an alternative to a “treat-all” or a “treat-none” strategy, patients can be classified into high-risk and low-risk groups on the basis of prognostic-factors for additional undetected metastases. Low-risk patients could be curatively treated, while high-risk groups could be treated as patients with poly-recurrences.

Validation of our model is essential before using our model for clinical decision making. Such validation effort seems worthwhile, as our results show that the use of prognostic subgroups for clinical decision making could potentially reduce incorrect assignment of curative or systemic treatment for patients without or with undetected metastases, compared to the “treat-all” or “treat-none” strategies (Table 5). The use of risk groups is likely to be both cost saving and beneficial for patients, as it may reduce unnecessary harmful treatments. A full cost–benefit analysis, including life expectancy, quality of life, and costs of the treatments, would be needed to confirm this assumption.

There are some patient subgroups in the model that have a relatively high chance to be assigned the wrong treatment. Ninety percent of the simulated patients had a very high or low risk of microscopic disease (above 80% or below 10%). A potential management strategy in the other ten percent could be to offer these patients more frequent surveillance, since the model predicted that 74% of patients with additional microscopic disease would be diagnosed as poly-recurrence within three months (Figure A2).

One feature not added to the model is death due to other causes than metastatic disease. Inclusion of death due to another cause would have reduced the benefit of curative treatment in oligo-recurrent disease. Other factors that should be taken into consideration for treatment decision making are the eligibility criteria of a patient for a certain therapy and the effects of a therapy on symptoms. Therefore, multidisciplinary teams and shared decision making are important to provide the best treatment for a specific patient. This simulation model is meant as a tool to support such a decision-making process. 

The microsimulation model and prognostic groups are not based on assumptions nor data of second primary lung cancers, synchronous oligo-metastases, or other types of cancer than NSCLC. Predictions of risk of undetectable metastases are therefore not necessarily applicable for these patients, although it is possible to collect data on the possible predictors, such as the sizes of the metastases in the clinical setting, and test if it is possible to create risk groups for those patients in a similar fashion. It can be informative and of great value for patients to collect clinical data to see if these conclusions are valid in those scenarios. Finally, once our prediction model for NSCLC is validated, the criteria of Martini and Melamed [45] should be applied to distinguish patients with potential oligo recurrences from patients with second primary lung cancers.

### 4.3. Microsimulation Model

Simulation modelling is a useful tool to synthesize the available evidence and extrapolate beyond currently observed data, especially in circumstances when relevant information and processes are either not available from a single source or unobservable. With respect to the growth of micro-metastatic lesions, there is a paucity in information that could support medical decision making. However, evidence from cell cultures [46], mouse models [47], and observed growth rates in larger tumours [26] provide indications about the tumour growth of micro-metastases in real patients. This information can be combined in a simulation model to allow inference on the disease aspects important for clinical or policy decisions. Although this microsimulation model is useful for improving insight into the metastatic process, and the selection of patients that may benefit from curative therapy, we need to keep in mind that all models are based on various assumptions. We have made an effort to be fully transparent by making all important model assumptions explicit and presenting the reasoning behind many of the choices made. Some model assumptions could be tested in the sensitivity analyses. However, validation of the model outcomes in clinical practice is still needed to determine if the model and its assumptions are correct.

### 4.4. Prognostic Groups

Prognostic factors have been identified and used to pool patients into risk groups. The 30% threshold value for low and high risk is arbitrary. Other thresholds may be used, and will influence the proportion of undetected metastases in the high- and low-risk groups. This threshold should therefore be in balance with the expected effects on quality of life and survival. Additional cost-effectiveness research can be used to determine the optimal threshold.

The diameter of the metastases was found to be the most important prognostic factor in this model. When building the microsimulation model, the focus was to make the model realistic with an appropriate level of complexity. The diameter predictor was an emergent property of the simulation model, which makes sense theoretically. The fact that the metastasis was invisible on the previous scan in combination with the size of the metastasis once detected, is informative about the growth rate of the metastases. A large metastasis therefore also has a high growth rate. Patients with fast-growing metastases have a relatively small chance of being detected as oligo+. 

The tumour volume can also have a negative impact on survival. For instance, Oh et al. reported a 1.04 death hazard ratio for each cm^3^ rise in volume of a brain metastasis [48]. However, in our model all metastases larger than 0.27 cm^3^ were considered to be large, but these metastases may still be classified as small in the model of Oh et al. Furthermore, the model outcomes are different: Oh et al. predict the chance to die from a specific metastasis, while our model predicts the risk of having additional metastases. The findings from the two models are, however, not contradictory, as patients with larger brain oligo-metastases may both have a greater hazard to die from their oligo-metastases and have a lower risk of additional metastases. Both models suggest that those patients could benefit from curative treatment.

Sensitivity analyses showed a high level of model robustness. Extreme parameter values resulted in minor effects on the ability to predict underlying metastases, and the outcomes still fall within a clinically observable range. Random variation of the parameters had little effect on model outcomes. Correlation between the growth rate and the numbers of metastases per patient improved the ability to distinguish between patients with and without additional undetected metastases. We expect that these features are associated; however, evidence for this hypothesis is lacking.

Using a different definition of oligo-metastatic disease, that is, detection of only one recurrence, affected the proportion of undetected metastases in the low-risk group. Extension of the definition to five recurrences did not increase the size of the low-risk group, but would have a negative effect on the relative number of unnecessary curative treatments if the “treat all” strategy was used. These findings match the literature in which the number of oligo-metastases per patient is also a prognostic factor [12].

When increasing the ability of metastases to metastasize, the size of the low-risk group diminishes accordingly. In the case that 80% of the patients have metastatic metastases, no low-risk patients with an indication for curative therapy remain identifiable. Although this model prediction is to be expected, it does not reflect the current clinical practice since there are patients that have long progression-free survival after curative treatment of oligo-metastases. Therefore, we argue that metastatic metastases may either be rare [49] or need more than 5 years to become detectable from the single-cell state. 

### 4.5. Future Developments

There have been intriguing developments in molecular diagnostics to identify microscopic tumour load, such as circulating tumour cells (CTCs) and circulating tumour DNA (ctDNA). Although insight into the true potential of these techniques requires continued research efforts, preliminary modelling studies have investigated its potential in the clinical setting. For example, the study by Coemans et al. [50] studied whether CTCs could be used to detect distant metastases before surgery of the primary tumour in breast cancer. Tang et al. show that ctDNA may be predictive for distinguishing “true” oligometastatic disease from poly recurrence, although they have a small sample size [51]. As soon as clinical studies are available that elucidate the relationship between ctDNA or CTCs and oligo recurrence, it will be interesting to include this information into our simulation model.

Symptom emergence is largely dependent on the location and size of the metastasis. The data on the location of the metastases is currently not of high enough quality to be included in this model. Future versions of this model could be made that includes the locations of the metastases, using location-specific hazard rates to give symptoms.

### 4.6. Recommendations on Data Collection

Most studies on curative treatment for oligo-metastases in NSCLC are either prospective, single-arm studies, or retrospective studies, which have a small sample size of patients or report below the number of parameters that are required for our research question. These studies are often based on highly selected patients with favourable inclusion criteria, using varying definitions of oligo-metastases. These studies are therefore susceptible to selection bias [17,52]. Two randomized studies have been reported with 29 and 49 enrolled patients [53,54]. Although the results show increased progression-free survival, strong evidence for a benefit of curative therapy for oligo-metastatic NSCLC is still absent.

In the absence of high-quality patient-level data, we have used microsimulation to provide guidance and generate new hypotheses. To allow validation and improvement or extension of the model, structural reporting of the tumour and patient features in a prospective manner would be highly valuable, preferably within the context of a randomized controlled trial investigating treatment for oligo-metastatic disease.

Currently, three phase 2 trials in oligo-metastatic disease, including lung cancer (NCT03905317, NCT03349203, NCT03965468), are ongoing. These studies, finishing in 2021, focus on the treatment of oligo-metastases and compare two treatments for that purpose. It is not clear whether the identification of prognostic and predictive factors is an additional goal of these studies, although it is possible that the studies’ data may be used for this purpose. Alternatively, the hypothesis of this model could also be tested in different types of cancer, such as colon cancer. To validate the model we present here, reporting on the diameters of the detected recurrences, the affected organs, the number of recurrences, and mode of detection (by symptoms or not, and which symptoms) is needed. To allow potential further improvement of the model, factors such as genetic markers, ctDNA and CTCs, histology of the primary tumour, or other prognostic factors [12] may be investigated for this specific purpose. These factors are not included in the current model.

## Figures and Tables

**Table 1:** Model input parameters.

Parameter	Value	Unit	Notes	Source
Simulated patients with metastases	100,000	patients	If 100,000 patients are simulated, 4.7% of patients (4708) are expected to have oligo-metastases.	Definition
Definition of oligo-recurrence	1–3	metastases	Varying definitions for the maximum number of metastases in oligo-recurrence have been used in literature. Most articles used as input for the model used this definition.	Definition
*M_total_*(*µ*,*σ*)	16, 34	metastases	Describes number of metastases per patient drawn from a normal distribution. Calibrated to proportion of oligo− (4.7%). (N = 880)	Calibration [6,7,8,20,21]
*t_FU_*	91, 182, 365, 548, 730, 1095, 1460, 1825	days	The surveillance schedule of the simulation model is constructed to match the surveillance of the patients in the Dutch cohorts as much as possible [24,25].	Definition
*σ_FU_*	15.5	days	To determine the time of the surveillance scan, a random normal variation around the planned scan time was used, with a 95% confidence interval of 1 month around the planned scan time. This value is based on expert opinion (L.A., E.A.K., S.Y.S., and F.M.N.H.S.).	Expert opinion
*t_FU min_*	61	days	No surveillance scans are planned before 2 months after curative treatment of the primary tumour. This value is based on expert opinion.	Expert opinion
*t_FU max_*	1825	days	The simulation and analysis stop after 5 years.	Definition
*λ_VDT_*	−0.006	days	Parameter is fitted to data with a negative exponential distribution, representing the distribution of VDTs between patients. (N = 415)	Literature [26,27,28,29]
*VDT_min_*	30	days^−1^	Cells require a minimum time to duplicate. Metastases with negative VDTs cannot pass the detection threshold, and therefore cannot affect the RFS.	Definition [30]
*VDT_max_*	365	days^−1^	A metastasis with a VDT of 365 days needs 36 years to reach the detection threshold and should rarely affect 5-year RFS. Expert opinion (HBW, RV, VMHC).	Expert opinion [30]
*V_det_*	0.07	cm^3^	Minimum detection diameter is set to 5 mm. Metastases of this size are assumed to be spherical.	Literature [31,32]
*R*(*α,β*)	9.1	-	Describes ratio of volumes of metastases per patient drawn from a beta distribution. Calibrated to the number of oligo-metastases detected derived from pooled average of patients (N = 1399).	Calibration [7,20,21]
*λ_detectable_*	0.00161	days^−1^	Calibrated to progression-free survival of curatively treated stage I NSCLC patients (N = 841).	Calibration [24,25]
*λ_symptom_*	0.00049	-	Hazard of a single metastasis becoming symptomatic. Calibrated to symptomatic detection rate of pooled average patients with detected recurrences (N = 393).	Calibration [33,34]
*p_mm_*	0.0576, 0.2606, 0.6656	-	Chance of the metastases becoming metastatic dependent on the total tumour volume. Calibrated to calculate a 20%, 50%, and 80% hazard. Only used in sensitivity analyses.	Calibration

**Table 2:** Description of the generated patient-level data (the median, with the 2.5% and 97.5% percentiles in brackets).

	*R*	VDT (days^−1^)	Diameter of the Largest Recurrence (cm)	Metastases Detected	Total Metastases	RFI (days)
All patients	0.93 (0.67–1.00)	103 (33–318)	0.58 (0.50–1.13)	6 (1–32)	21 (2–49)	510 (74–1772)
Poly metastases	0.95 (0.75–1.00)	88 (33–297)	0.61 (0.51–1.21)	10 (4–35)	22 (5–49)	542 (75–1777)
Oligo+	0.85 (0.59–0.98)	146 (38–337)	0.53 (0.50–0.66)	2 (1–3)	22 (5–49)	362 (71–1579)
Oligo−	0.92 (0.67–1.00)	104 (34–319)	0.59 (0.50–1.59)	2 (1–3)	2 (1–3)	530 (77–1817)
High-risk group	0.86 (0.60–0.99)	143 (38–336)	0.53 (0.50–0.69)	2 (1–3)	20 (1–49)	363 (72–1629)
Low-risk group	0.91 (0.47–1.00)	55 (32–138)	0.99 (0.81–2.95)	2 (1–3)	2 (1–25)	1075 (192–1825)

VDT, volume doubling time; RFI, recurrence-free interval; Symptomatic, symptomatic (unscheduled) detection.

**Table 3:** Odds Ratios predicted by the logistic regression model.

Predictor	Odds Ratio
1 Metastasis detected	reference
2 Metastases detected	1.76
3 Metastases detected	2.44
Asymptomatic detection	reference
Symptomatic detection	1.63
Small size (<6 mm)	reference
Medium size (6–8 mm)	6.90
Large size (>8 mm)	146.79

**Table 4:** Proportion of oligo+ within patients with detected oligo-metastases in the Base Case scenario.

	Asymptomatic			Symptomatic		
Metastases detected:	1	2	3	1	2	3
Small (<6 mm)	0.91	0.92	0.93	0.88	0.88	0.83
Medium (6–8 mm)	0.08	0.61	0.81	0.08	0.71	0.84
Large (>8 mm)	0.00	0.02	0.19	0.00	0.00	0.24

Proportions of oligo+ per risk sub-group. Grey fields represent the low-risk group.

**Table 5:** Performance of the risk-group-based treatment selection compared to “treat-all” and “treat-none” strategies.

Strategy	Low Risk		High Risk		Performance of Chosen Strategy (%)				
	Oligo−	Oligo+	Oligo−	Oligo+	Sensitivity	Specificity	PPV	NPV	Accuracy
Risk groups	1252	111	3427	28,716	26.8	99.6	91.9	89.3	89.4
Treat-all	4691	28,815	0	0	100.0	0.0	14.0	0.0	14.0
Treat-none	0	0	4691	28,815	0.0	100.0	0.0	86.0	86.0

For calculation of the strategy performance, low-risk oligo− is a true positive, low-risk oligo+ is a false positive, high-risk oligo− is a false negative, and high-risk oligo+ is true negative. PPV, positive predictive value; NPV, negative predictive value.

**Table 6:** Proportion of detected oligo-metastases that fall into the risk groups and proportion of oligo+ within the risk groups per scenario.

Sensitivity Analysis Scenario	All Oligo-Metastases at Time of Detection		Low Risk		High Risk	
	N	% Oligo+	% of All	% Oligo+	% of All	% Oligo+
Base Case	33,506	86.0	4.1	8.1	95.9	89.3
*M_total_* Lower	32,194	91.0	2.7	11.2	97.3	93.5
*M_total_* Upper	34,506	82.0	5.1	6.2	94.9	86.3
RFS Lower	32,600	85.0	4.5	7.6	95.5	88.9
RFS Upper	34,597	86.0	3.8	8.4	96.2	89.5
*R* Lower	28,239	83.0	4.6	1.1	95.4	87.3
*R* Upper	35,992	87.0	3.9	14.5	96.1	90.2
Symptomatic Detection Lower	32,917	86.0	4.2	6.3	95.8	89.2
Symptomatic Detection Upper	34,390	86.0	4.0	7.4	96.0	89.7
Random Detection Size Scan	33,641	86.0	3.9	9.1	96.1	89.2
Random *VDT*	33,721	86.0	4.2	8.9	95.8	89.0
Correlation *ρ* = 0.5	27,879	83.0	7.4	6.8	92.6	89.7
Correlation *ρ* = 1.0	23,727	80.0	13.2	4.6	86.8	91.8
Definition of oligo-recurrence = 1 *	12,757	89.0	5.6	5.7	94.4	93.8
Definition of oligo-recurrence = 5 *	47,802	82.0	2.9	7.9	97.1	84.5
Metastatic metastases 20% *	33,506	87.2	2.1	15.1	97.9	88.8
Metastatic metastases 50% *	33,506	89.2	1.4	21.1	98.6	90.2
Metastatic metastases 80% *	33,506	92.4	0.0	-	100	92.4

* = Risk groups are made up of different subgroups compared to the Base Case.

## Data Availability

Data available in a publicly accessible repository that does not issue DOIs. Publicly available software was used in this study. This software can be found here: [https://github.com/HaroldWolff/RecurrenceSimulation (accessed on 7 April 2021)].

## Appendix A

### Appendix A.1. Derivation of Function (4)

*m_det_* is the number of detected metastases and is per definition an integer number between 0 and *M_total_*. The number of detected metastases is equal to the number of metastases of detectable size (*V_det_*) at the time that a scan occurs (*t_scan_*). Metastasis *i* reaches detectable size (*V_det_*) at *t*_det *i*_; thus, *i* metastases are detectable when $t_{\det i+1}\geq t_{scan}\geq t_{\det i}$. Furthermore, the detection threshold should also fall in between *V_i_* and *V_i_*_+1_ at the time of this scan: $V_{i+1}\left(t_{scan}\right)\leq V_{det}\leq V_{i}\left(t_{scan}\right)$. mdet can be obtained by solving *i*.

Applying Function (1) on the formula above results in:

(A1) $$V_{i+1}\left(0\right)\;\times \;2^{t_{scan}/VDT}\leq V_{0}\left(0\right)\;\times \;2^{t_{\det0}/VDT}\leq V_{i}\left(0\right)\;\times \;2^{t_{scan}/VDT}$$

And according to Function (2):

(A2) $$V_{0}\left(0\right)\;\times \;R^{i+1}\;\times \;2^{t_{scan}/VDT}\leq V_{0}\left(0\right)\;\times \;2^{t_{\det0}/VDT}\leq V_{0}\left(0\right)\;\times \;R^{i}\;\times \;2^{t_{scan}/VDT}$$

This can be simplified to: $i+1\leq \frac{\left(t_{\det0}-t_{scan}\right)}{VDT\times log_{2}\left(R\right)}\leq i$.

*m_det_* can be obtained by rounding *i* to an integer number: $\lceil \frac{\left(t_{\det0}-t_{scan}\right)}{VDT\times log_{2}\left(R\right)}\rceil =m_{det}$.

### Appendix A.2. Sensitivity Analyses: Alternative Calibration Targets

The accuracy of the risk groups was tested by generating a new patient database using the regular prediction model with extreme parameters.

For each of the four model parameters that were obtained by calibration, one by one, the upper and lower limit of the 95% confidence intervals around the calibration targets were used to recalibrate the model. For the pooled averages of the rates (*µ*) from the literature (for oligo-metastases detected, oligo-, and symptomatic detections) the 95% confidence interval around this rate was estimated to be $\mu \pm 1.96\sqrt{\frac{\mu \;\left(1-\mu \right)}{n}}$. These recalibrated microsimulation models were run again, and the risk sub-group analyses were repeated. All the other model parameters were left unchanged.

### Appendix A.3. Sensitivity Analyses: Random Normal Variation around the Model Parameters

In the second set of sensitivity analyses, the parameters *VDT* and *V_det_* were separately varied around the point estimates by randomly drawing a new value from a normal distribution per patient. The scan detection threshold was altered around 5 mm with a standard deviation of 1 mm. The individually drawn volume doubling time was altered for the smallest detectable metastases with a standard deviation of 10 days. The microsimulation model was run again, and the risk sub-group analyses were repeated. All the other model parameters were left unchanged.

### Appendix A.4. Sensitivity Analyses: Correlation between Volume Doubling Time and the Total Number of Metastases

In the Base Case model, no interactions were assumed between model parameters, because proof for such interactions was lacking. However, it is a credible assumption to make that patients with higher VDTs also have higher numbers of metastases. We explored the effect of such an interaction by adding a correlation between *VDT* and *M_total_* using the formula:

(A3) $$VDT=\frac{\ln\left(\rho \;\times \;\frac{\left(M_{total}-\mu_{M_{total}}\right)}{\sigma_{M_{total}}}+\left(1-\rho \right)\;\times \;r\right)}{\lambda_{VDT}}$$

with *ρ* as the “correlation coefficient”, and *r* as a random uniform value that is drawn to determine the *VDT* of a patient’s metastases. Two simulations were used for the sensitivity analyses, with *ρ* = 0.5 and *ρ* = 1.0. The microsimulation model was run again, and the risk sub-group analyses were repeated. All the other model parameters were left unchanged.

### Appendix A.5. Sensitivity Analyses: Adaptation of the Definition of Oligo-Metastases

For these sensitivity analyses, the simulated patient-level database was unaltered, but the prediction model was modified by setting the maximum number of detected recurrences that is considered to be an oligo-metastasis Moligo max to 1 and to 5. This change in definition alters the number of patients with detected oligo-metastases and the proportions of oligo+ and oligo− within the risk sub-groups.

### Appendix A.6. Sensitivity Analyses: Metastasizing Metastases

A sensitivity analysis was included that estimates the impact of the unverified assumption that both oligo and poly metastases are able to produce additional metastases, without the presence of the primary tumour. This increases the proportion of oligo+. For this sensitivity analysis, we assumed that the mutations required for the metastasis to metastasize are related to the number of DNA duplication steps and cell divisions (A4). Therefore, we assume that the hazard of metastases acquiring the ability to metastasize is related to the total tumour volume in time:

(A4) $$Hazard=1-{\left(1-p_{mm}\right)}^{V_{total}\left(t\right)}$$

Here, *p_mm_* is the probability that one metastatic cell within the total tumour volume will acquire the ability to disseminate metastases, and *V_total_*(*t*) is the total tumour volume in time. The hazard was used to estimate how many of the patients with oligo-metastases in the model have developed additional metastases after removal of the primary tumour. *p_mm_* was calibrated to create scenarios with 20%, 50%, and 80% metastatic metastases (Table 1). The proportions of oligo+ per risk sub-group was subsequently adjusted for patients that switched from oligo− to oligo+.

**Table A1:** Proportion of oligo+ within patients with detected oligo-metastases in the *M_total_* Lower scenario.

	Asymptomatic			Symptomatic		
Metastases detected:	1	2	3	1	2	3
Small (<6 mm)	0.95	0.95	0.96	0.91	0.91	0.89
Medium (6–8 mm)	0.12	0.76	0.87	0.16	0.76	0.86
Large (>8 mm)	0.00	0.02	0.25	0.00	0.06	0.27

Proportions of oligo+ per risk sub-group. Grey fields represent the low-risk group.

**Table A2:** Proportion of oligo+ within patients with detected oligo-metastases in the *M_total_* Upper scenario.

	Asymptomatic			Symptomatic		
Metastases detected:	1	2	3	1	2	3
Small (<6 mm)	0.88	0.90	0.91	0.83	0.83	0.78
Medium (6–8 mm)	0.06	0.58	0.77	0.07	0.58	0.73
Large (>8 mm)	0.00	0.02	0.17	0.00	0.00	0.11

Proportions of oligo+ per risk sub-group. Grey fields represent the low-risk group.

**Table A3:** Proportion of oligo+ within patients with detected oligo-metastases in the RFS Lower scenario.

	Asymptomatic			Symptomatic		
Metastases detected:	1	2	3	1	2	3
Small (<6 mm)	0.91	0.92	0.91	0.89	0.86	0.83
Medium (6–8 mm)	0.08	0.64	0.83	0.10	0.55	0.77
Large (>8 mm)	0.00	0.02	0.17	0.00	0.06	0.19

Proportions of oligo+ per risk sub-group. Grey fields represent the low-risk group.

**Table A4:** Proportion of oligo+ within patients with detected oligo-metastases in the RFS Upper scenario.

	Asymptomatic			Symptomatic		
Metastases detected:	1	2	3	1	2	3
Small (<6 mm)	0.91	0.92	0.93	0.88	0.88	0.84
Medium (6–8 mm)	0.10	0.63	0.81	0.14	0.69	0.82
Large (>8 mm)	0.00	0.04	0.18	0.00	0.00	0.03

Proportions of oligo+ per risk sub-group. Grey fields represent the low-risk group.

**Table A5:** Proportion of oligo+ within patients with detected oligo-metastases in the *R* Lower scenario.

	Asymptomatic			Symptomatic		
Metastases detected:	1	2	3	1	2	3
Small (<6 mm)	0.90	0.91	0.92	0.85	0.82	0.79
Medium (6–8 mm)	0.01	0.40	0.69	0.00	0.41	0.69
Large (>8 mm)	0.00	0.00	0.03	0.00	0.00	0.05

Proportions of oligo+ per risk sub-group. Grey fields represent the low-risk group.

**Table A6:** Proportion of oligo+ within patients with detected oligo-metastases in the *R* Upper scenario.

	Asymptomatic			Symptomatic		
Metastases detected:	1	2	3	1	2	3
Small (<6 mm)	0.92	0.93	0.93	0.87	0.86	0.85
Medium (6–8 mm)	0.18	0.71	0.84	0.04	0.68	0.81
Large (>8 mm)	0.00	0.05	0.28	0.00	0.07	0.22

Proportions of oligo+ per risk sub-group. Grey fields represent the low-risk group.

**Table A7:** Proportion of oligo+ within patients with detected oligo-metastases in the Symptomatic Detection Lower scenario.

	Asymptomatic			Symptomatic		
Metastases detected:	1	2	3	1	2	3
Small (<6 mm)	0.91	0.92	0.93	0.86	0.86	0.83
Medium (6–8 mm)	0.05	0.61	0.79	0.17	0.68	0.81
Large (>8 mm)	0.00	0.01	0.17	0.00	0.00	0.11

Proportions of oligo+ per risk sub-group. Grey fields represent the low-risk group.

**Table A8:** Proportion of oligo+ within patients with detected oligo-metastases in the Symptomatic Detection Upper scenario.

	Asymptomatic			Symptomatic		
Metastases detected:	1	2	3	1	2	3
Small (<6 mm)	0.91	0.93	0.93	0.88	0.87	0.84
Medium (6–8 mm)	0.07	0.65	0.82	0.13	0.68	0.81
Large (>8 mm)	0.00	0.01	0.20	0.00	0.03	0.18

Proportions of oligo+ per risk sub-group. Grey fields represent the low-risk group.

**Table A9:** Proportion of oligo+ within patients with detected oligo-metastases in the Random Detection Size Scan scenario.

	Asymptomatic			Symptomatic		
Metastases detected:	1	2	3	1	2	3
Small (<6 mm)	0.91	0.92	0.92	0.84	0.89	0.84
Medium (6–8 mm)	0.10	0.66	0.80	0.07	0.71	0.80
Large (>8 mm)	0.00	0.02	0.22	0.00	0.03	0.25

Proportions of oligo+ per risk sub-group. Grey fields represent the low-risk group.

**Table A10:** Proportion of oligo+ within patients with detected oligo-metastases in the Random *VDT* scenario.

	Asymptomatic			Symptomatic		
Metastases detected:	1	2	3	1	2	3
Small (<6 mm)	0.90	0.92	0.93	0.89	0.86	0.83
Medium (6–8 mm)	0.11	0.64	0.80	0.00	0.68	0.83
Large (>8 mm)	0.00	0.02	0.19	0.00	0.05	0.18

Proportions of oligo+ per risk sub-group. Grey fields represent the low-risk group.

**Table A11:** Proportion of oligo+ within patients with detected oligo-metastases in the Correlation *ρ* = 0.5 scenario.

	Asymptomatic			Symptomatic		
Metastases detected:	1	2	3	1	2	3
Small (<6 mm)	0.93	0.93	0.94	0.94	0.90	0.85
Medium (6–8 mm)	0.10	0.65	0.80	0.15	0.57	0.81
Large (>8 mm)	0.00	0.02	0.14	0.00	0.00	0.11

Proportions of oligo+ per risk sub-group. Grey fields represent the low-risk group.

**Table A12:** Proportion of oligo+ within patients with detected oligo-metastases in the Correlation *ρ* = 1.0 scenario.

	Asymptomatic			Symptomatic		
Metastases detected:	1	2	3	1	2	3
Small (<6 mm)	0.95	0.95	0.96	0.93	0.92	0.90
Medium (6–8 mm)	0.11	0.69	0.83	0.15	0.64	0.86
Large (>8 mm)	0.00	0.01	0.09	0.00	0.00	0.09

Proportions of oligo+ per risk sub-group. Grey fields represent the low-risk group.

**Table A13:** Proportion of oligo+ within patients with detected oligo-metastases in the redefinition of oligo metastases to 1 metastasis scenario.

	Asymptomatic	Symptomatic
Metastases detected:	1	1
Small (<6 mm)	0.94	0.89
Medium (6–8 mm)	0.09	0.08
Large (>8 mm)	0.00	0.00

Proportions of oligo+ per risk sub-group. Grey fields represent the low-risk group. This scenario has fewer sub-groups than the Base Case.

**Table A14:** Proportion of oligo+ within patients with detected oligo-metastases in the redefinition of oligo metastases to 1–5 metastases scenario.

	Asymptomatic					Symptomatic				
Metastases detected:	1	2	3	4	5	1	2	3	4	5
Small (<6 mm)	0.87	0.88	0.90	0.90	0.90	0.84	0.84	0.80	0.79	0.74
Medium (6–8 mm)	0.08	0.58	0.78	0.81	0.85	0.08	0.70	0.80	0.73	0.69
Large (>8 mm)	0.00	0.02	0.18	0.39	0.52	0.00	0.00	0.22	0.51	0.60

Proportions of oligo+ per risk sub-group. Grey fields represent the low-risk group. This scenario has more sub-groups than the Base Case.

**Table A15:** Proportion of oligo+ within patients with detected oligo-metastases in the Metastatic metastases 20% scenario.

	Asymptomatic			Symptomatic		
Metastases detected:	1	2	3	1	2	3
Small (<6 mm)	0.91	0.92	0.94	0.88	0.88	0.84
Medium (6–8 mm)	0.12	0.64	0.83	0.14	0.73	0.85
Large (>8 mm)	0.21	0.34	0.48	0.16	0.32	0.46

Proportions of oligo+ per risk sub-group. Grey fields represent the low-risk group. This scenario has fewer categories that are considered low risk than the Base Case.

**Table A16:** Proportion of oligo+ within patients with detected oligo-metastases in the Metastatic metastases 50% scenario.

	Asymptomatic			Symptomatic		
Metastases detected:	1	2	3	1	2	3
Small (<6 mm)	0.92	0.93	0.95	0.89	0.89	0.86
Medium (6–8 mm)	0.21	0.70	0.87	0.26	0.77	0.88
Large (>8 mm)	0.46	0.64	0.73	0.45	0.60	0.73

Proportions of oligo+ per risk sub-group. Grey fields represent the low-risk group. This scenario has fewer categories that are considered low risk than the Base Case.

**Table A17:** Proportion of oligo+ within patients with detected oligo-metastases in the Metastatic metastases 80% scenario.

	Asymptomatic			Symptomatic		
Metastases detected:	1	2	3	1	2	3
Small (<6 mm)	0.93	0.95	0.96	0.90	0.91	0.90
Medium (6–8 mm)	0.40	0.81	0.93	0.45	0.86	0.93
Large (>8 mm)	0.74	0.89	0.93	0.77	0.85	0.93

Proportions of oligo+ per risk sub-group. This scenario has no low-risk categories in contrast to the Base Case.
